# A Preliminary Study of the Effects of Attentive Music Listening on Cochlear Implant Users’ Speech Perception, Quality of Life, and Behavioral and Objective Measures of Frequency Change Detection

**DOI:** 10.3389/fnhum.2020.00110

**Published:** 2020-03-31

**Authors:** Gabrielle M. Firestone, Kelli McGuire, Chun Liang, Nanhua Zhang, Chelsea M. Blankenship, Jing Xiang, Fawen Zhang

**Affiliations:** ^1^Department of Communication Sciences and Disorders, University of Cincinnati, Cincinnati, OH, United States; ^2^Division of Biostatistics and Epidemiology, Cincinnati Children’s Hospital Medical Center, Cincinnati, OH, United States; ^3^Department of Pediatrics, University of Cincinnati College of Medicine, Cincinnati, OH, United States; ^4^Department of Pediatrics and Neurology, Cincinnati Children’s Hospital Medical Center, Cincinnati, OH, United States

**Keywords:** cochlear implant, music training, frequency change detection, electroencephalogram, cortical auditory evoked potential

## Abstract

**Introduction:**

Most cochlear implant (CI) users have difficulty in listening tasks that rely strongly on perception of frequency changes (e.g., speech perception in noise, musical melody perception, etc.). Some previous studies using behavioral or subjective assessments have shown that short-term music training can benefit CI users’ perception of music and speech. Electroencephalographic (EEG) recordings may reveal the neural basis for music training benefits in CI users.

**Objective:**

To examine the effects of short-term music training on CI hearing outcomes using a comprehensive test battery of subjective evaluation, behavioral tests, and EEG measures.

**Design:**

Twelve adult CI users were recruited for a home-based music training program that focused on attentive listening to music genres and materials that have an emphasis on melody. The participants used a music streaming program (i.e., Pandora) downloaded onto personal electronic devices for training. The participants attentively listened to music through a direct audio cable or through Bluetooth streaming. The training schedule was 40 min/session/day, 5 days/week, for either 4 or 8 weeks. The pre-training and post-training tests included: hearing thresholds, Speech, Spatial and Qualities of Hearing Scale (SSQ12) questionnaire, psychoacoustic tests of frequency change detection threshold (FCDT), speech recognition tests (CNC words, AzBio sentences, and QuickSIN), and EEG responses to tones that contained different magnitudes of frequency changes.

**Results:**

All participants except one finished the 4- or 8-week training, resulting in a dropout rate of 8.33%. Eleven participants performed all tests except for two who did not participate in EEG tests. Results showed a significant improvement in the FCDTs as well as performance on CNC and QuickSIN after training (*p* < 0.05), but no significant improvement in SSQ scores (*p* > 0.05). Results of the EEG tests showed larger post-training cortical auditory evoked potentials (CAEPs) in seven of the nine participants, suggesting a better cortical processing of both stimulus onset and within-stimulus frequency changes.

**Conclusion:**

These preliminary data suggest that extensive, focused music listening can improve frequency perception and speech perception in CI users. Further studies that include a larger sample size and control groups are warranted to determine the efficacy of short-term music training in CI users.

## Introduction

A cochlear implant (CI) is a prosthetic device which allows individuals with severe-to-profound hearing loss to hear sounds. CIs collect, process, and convert sounds into electrical signals, which are used to directly stimulate the auditory nerve through the electrode array inserted into the cochlea. Although CI usage typically allows for satisfactory speech perception in quiet, CI users’ performance in tasks that heavily rely on pitch perception is typically poor. Such tasks include speech perception in noise that requires the differentiation of the voice fundamental frequency (F0) between target and competing voices, and music melodic perception that requires the detection of dynamic pitch changes (Limb, 2006; Cullington and Zeng, 2008; Oxenham, 2008; Cousineau et al., 2010).

Deficits in pitch perception have been shown to limit CI outcomes. Previous studies have used frequency discrimination or pitch ranking tasks, in which participants are required to identify the target frequency that is different from the reference frequency or to determine which of the presented sounds is higher in pitch. These studies have shown that CI users’ ability to discriminate frequencies is significantly correlated with speech performance (Kenway et al., 2015; Turgeon et al., 2015). Because speech or melody perception requires the ability to detect dynamic frequency changes or relative pitch (McDermott and Oxenham, 2008), it is important to examine CI users’ ability to detect frequency changes contained within a stimulus. Our lab first reported that CI users’ frequency change detection thresholds (FCDTs; the minimum frequency change within a stimulus that can be detected by an individual) were significantly correlated to speech performance, further supporting the important role of frequency change detection ability in CI outcomes (Zhang et al., 2019).

The poor performance of pitch change detection in CI users can be largely attributed to CI technological constraints (poor frequency resolution due to limited number of electrodes and the removal of fine temporal structure information by the CI signal processing). Even with speech coding strategies that provide temporal fine structure of the signal, CI users generally do not seem to benefit from it (Riss et al., 2011). Additionally, other factors that may limit pitch change detection include neural-electrode interface (e.g., the distance between the electrode and neural elements), neural deficits related to sound deprivation, and cognitive function decline (Di Nardo et al., 2010; Limb and Roy, 2014; Pisoni et al., 2018).

Although CI users face the aforementioned technological and biological constraints, their hearing may be significantly improved with auditory training (Fu and Galvin, 2007), which may enhance the sensitivity of the auditory system to detect sounds through training-induced brain plasticity (Irvine, 2018). Music training may have some advantages over other auditory training approaches (e.g., speech training) in terms of cross-domain auditory plasticity because music training enhances overlapping brain networks shared by language and music processing (Besson et al., 2007). Music perception may also place higher demands on auditory processing than does speech perception as it engages emotion and focused attention, which are important for speech perception (Patel, 2014; Lehmann and Skoe, 2015). Such cross-domain plasticity may significantly impact CI users who undergo auditory training to improve both speech and music perception.

Numerous studies involving normal-hearing listeners have reported positive effects of music training on pitch perception. For instance, our group and other researchers have reported that musicians, who undergo years of music training, exhibit superior performance of pitch perception and better neurophysiological responses relative to non-musicians (Besson et al., 2007; Kraus et al., 2009; Itoh et al., 2012; Fuller et al., 2014; Brown et al., 2017; Liang et al., 2018). Such long-term music training may be expanded to the speech domain and positively affect speech perception in noise (Parbery-Clark et al., 2009; Baskent and Gaudrain, 2016). These differences in neural responses and auditory function in musicians vs. non-musicians are more likely to arise from training-induced brain plasticity rather than from their pre-existing biological predispositions for music (Besson et al., 2007). Short-term music training in normal hearing listeners can also result in improved performance in speech perception and neuroplastic changes (Lappe et al., 2008; Jain et al., 2015; Nan et al., 2018).

Music training may seem questionable in CI users, as current CIs provide little to no harmonic information (important for musical pitch and timbre perception) and music generally does not sound pleasant for many CI users. As a result, post-lingually deafened adult CI users, who can compare sound quality to experience before implantation or hearing loss, are much less likely to expose themselves to music (Limb, 2006; Oxenham, 2008; Looi and She, 2010). Moreover, music perception has been given less weight than speech perception in terms of CI outcomes, evidenced by the lack of assessment in music perception in most clinical settings. The argument for music training in CI users is that it may enhance both music and speech outcomes of the CI, while training with only speech stimuli has not been reported to enhance perception in non-speech domains. While CI manufacturers and some researchers have advocated using musical stimuli for post-implantation rehabilitation (Gfeller et al., 2015; Hutter et al., 2015), there is a need for evidence-based training approaches for clinical rehabilitation.

Previous research examining short-term music training have reported positive hearing outcomes in CI users (see review by Gfeller, 2016). For instance, training on melodic contour identification or pitch patterns has been shown to improve CI users’ sensitivity to pitch pattern perception, and/or music and speech perception (Galvin et al., 2009; Fu et al., 2015; Lo et al., 2015; Smith et al., 2017; Cheng et al., 2018). Other music training approaches (e.g., playing, singing, listening to music) can also improve music and speech perception (Gfeller et al., 2015; Good et al., 2017). Note that these music training studies have mainly used computer-based programs for training and subjective and behavioral measures for assessing the training effects.

There are only two neurophysiological studies that examined music training effects in CI users. One study used the positron emission topography (PET; Petersen et al., 2009) technique to examine the outcomes of a music-ear training program (singing, playing, and listening) during the first several months of CI use. The authors only reported the preliminary results from one CI user, showing that the activation in temporal lobes enlarged over time. The other study recorded the mismatch negativity (MMN) from CI users using the electroencephalographic (EEG) technique before and after a 2-week music program (a combination of active music-making and computer-based music listening) that focused on strengthening the participants’ perception of music pitch, rhythm, and timbre (Petersen et al., 2015). The authors did not find a significant effect of music training on the MMN, which may be due to the brevity of the training program, the limitation of the MMN (e.g., small amplitude), and/or the lack of music training effect on the cortical processing of sound differences reflected by the MMN in CI users.

There is a need for understanding the fundamental neural basis of music training effects in CI users, which is critical for future efforts in developing music training programs and objective tools to monitor training progress and determine training efficacy. Using the EEG technique, one can record the cortical auditory evoked potentials (CAEPs), which reflect automatic cortical processing of sounds. CAEP recording does not require the subject’s voluntary response to the stimuli and thus is a valuable tool to assess cortical processing of sounds in difficult-to-test patients.

Cortical auditory evoked potentials can be evoked by the stimulus onset (onset CAEP) and a change within a stimulus (acoustic change complex or ACC, Friesen and Tremblay, 2006; Martin, 2007; Mathew et al., 2017; Liang et al., 2018). Unlike the onset CAEP that reflects cortical detection of stimulus onset, the ACC reflects a listener’s cortical processing of acoustic changes and it is corresponding to auditory discrimination ability (He et al., 2012; Kim, 2015; Brown et al., 2017). Compared to the MMN, the ACC is more time-efficient (every stimulus contributes to the ACC and thus a smaller number of stimulus trials are required), has a larger and more stable amplitude, and has better test–retest reliability (Kim, 2015).

The present study used comprehensive assessment methods including subjective evaluation, behavioral tests, and EEG to examine the effects of music training on hearing outcomes in CI users. The onset CAEPs and ACC data were used to evaluate how music training reshapes the auditory system.

## Materials and Methods

### Subjects

Twelve adult CI users (age range: 31–77 years; mean: 54.8 years) with severe-to-profound bilateral sensorineural hearing loss were recruited for music training. One participant was lost to follow-up after pre-training test (due to home relocation), resulting in a dropout rate of 8.33%. The demographic information for all 11 participants who finished the music training is shown in Table 1.

**TABLE 1:** Cochlear implant (CI) users’ demographics.

CI ear ID	Gender	Ear tested	Age	Type of CI user	Type of CI	Age at implantation	Duration of deafness before CI (years)	Duration of CI use (years)	Etiology
S1	F	L	64	Unilateral	Nucleus 5	58	45	6	Ototoxicity
S2	M	R	43	Bilateral	Hybrid*	39	4	4	Possibly ototoxicity
S3	M	R	31	Bilateral	Nucleus 5	21	0.20	10	Head trauma
S4^$^	F	R	50	Bilateral	Nucleus 6	43	39	7	Measles
S5^$^	F	R	50	Bilateral	Nucleus 6	42	40	8	Unknown
S6	M	L	64	Bilateral	Nucleus 6	59	8	6	Noise exposure
S7	F	R	47	Unilateral	Nucleus 6	45	17	2	Unknown
S8	M	R	59	Unilateral	Nucleus 6	48	15	12	Possibly genetic
S9^$^	M	L	44	Unilateral	Nucleus 5	35	35	9	Unknown
S10^#^	F	R	77	Bilateral	Nucleus 6	71	1.5	6	Unknown
S11^#^	M	R	76	Bilateral	Nucleus 6	73	56	3	Unknown

*This participant was implanted with a Hybrid electrode array, which was clinically mapped as a standard electrode array. ^#^Participants only have non-EEG data. EEG tests were not performed due to the EEG lab relocation. ^$^Participants are pre-lingually deafened.

Of the 11 subjects, 4 were unilateral and 7 were bilateral CI users, 3 were pre-lingually and 8 were post-lingually deafened. One CI recipient was implanted with a Hybrid electrode array however due to the loss of low-frequency hearing shortly following implantation, it was clinically mapped as a standard electrode array. All participants except one (S3) had used a hearing aid before cochlear implantation. All participants were native English speakers, had used their CIs for at least 1 year, had no psychological disorders, and had not received any music training pre- or post-implantation. All participants wore Cochlear America devices. Each participant completed music training in only one ear. For bilaterally implanted subjects, the poorer ear was subjectively selected by the participant and verified by the experimenter using behavioral data collected prior to the music training and the CI on the non-trained ear was switched off. Each subject provided written informed consent before participating, and all received monetary compensation. This research study was approved by the Institutional Review Board of the University of Cincinnati.

### Procedures

The measures used to assess music training effects include a questionnaire, audiometric thresholds, FCDTs, speech perception scores, and EEG recordings. All 11 participants who completed all pre- and post-training measures, except for two participants who were unable to complete the EEG tests due to the relocation of our EEG lab. Except for the questionnaire, all tests were conducted in randomized order inside a double-walled sound treated booth. Acoustic stimuli were presented via a loudspeaker in the sound field located approximately 4 ft from the patient at head height, at 0-degree azimuth, and at the most comfortable loudness level. CI users were allowed to adjust their processor sensitivity setting to the most comfortable setting, which was kept the same at pre- and post-training testing. Only the ear being trained was tested. For the non-test ear, the ear was either plugged with a foam earplug in unilateral CI users or the CI processor was off in bilateral CI users. During the study, there was no change in the CI mapping of the participants.

#### Speech Spatial and Qualities of Hearing Scale (SSQ12)

The Speech, Spatial, and Qualities of Hearing Scale (SSQ) questionnaire is a subjective measure of hearing that requires the participant to rate hearing abilities in different listening situations. Questions include the individual’s ability to hear in situations such as speech in noise, the direction and location of sounds, sound segregation, quality and naturalness of sound, and ease of listening. The SSQ12 is a small-scale, quicker version of the SSQ49, while still providing a comparable score to the original test (Noble et al., 2013). The total score ranges from 0 to 10, with 10 being the highest. The average score for the 12 questions was obtained for each participant.

#### Audiometry

Hearing thresholds were measured using pulsed tones in the range of 0.25–6 kHz (0.25, 0.5, 1, 2, 3, 4, and 6 kHz) using the Grason-Stadler (GSI-AudioStar Pro) audiometer, with a 5-dB as the minimum step size for intensity change. This would ensure audibility of sound presented through their CI processors.

#### Frequency Change Detection Test

All stimuli were 1 s in duration, with an initial frequency of 0.25, 1, or 4 kHz for the first 0.5 s followed by a higher frequency for the remaining 0.5 s. The frequency change from the base frequency ranged from 0.25 to 200% to accommodate the expected wide range of FCDTs in CI participants. The frequency change occurred for an integer number of base frequency cycles (zero crossing) to prevent audible transient clicks (Dimitrijevic et al., 2008). Amplitudes of all stimuli were equalized.

Psychoacoustic testing was administered using Angel Sound software^1^. An adaptive, 3-alternative forced-choice (3AFC) procedure was used to measure FCDTs, defined as the minimum detectible change in frequency. Each trial consisted of a target stimulus that contained the frequency change and two standard stimuli with no frequency change; the order of standard and target stimuli was randomized across trials. The inter-stimulus-interval was 0.5 s. During testing, the participant was asked to choose the different stimulus by pressing the response button on the computer screen. The initial frequency change of the target stimulus was 18% above the base frequency; subsequent frequency change step sizes were adjusted according to participants’ response. A 2-down 1-up staircase technique was used to target the 79% correct point on the psychometric function. For each base frequency, FCDT was averaged across the final six reversals in frequency change steps. The order of the 3 base frequency conditions was randomized across participants. For both pre- and post-training testing, FCDTs were measured twice to examine test–retest reliability to observe potential procedural learning effects.

#### Speech Perception Tests

##### Consonant-nucleus-consonant (CNC)

The CNC test is part of the Minimum Speech Test Battery (MSTB) that has been recommended by clinicians and researchers to evaluate word recognition in quiet in adult CI users (Gifford et al., 2015). In this study, one 50-word CNC list was administered to each participant. Different word lists were given at the pre- and post-training testing in order to eliminate any memory contribution to the results. The results were expressed as word scores (percent correct).

##### Arizona biomedical sentence recognition test (AzBio)

The AzBio Sentences (Spahr et al., 2012) are commonly used to measure sentence recognition performance in previous CI studies (e.g., Gifford et al., 2015). One 20-sentence list was presented in quiet and the participants were instructed to repeat each sentence that they heard and to guess if they were unsure. Results were manually recorded by the experimenter and expressed as percent correct (total words repeated correctly/total words presented).

##### Quick speech-in-noise (QuickSIN) test

QuickSIN was used to assess the ability to perceive speech in noise. The QuickSIN is more sensitive at measuring performance of speech in noise than the BKB-SIN or the HINT (Wilson et al., 2007). There are 12 lists of six sentences, each possessing five keywords that must be repeated by the subject to receive a score. The signal-to-noise (SNR) ratio starts at +25 in the first sentence and then decreases to 0 in 5-dB steps over the course of the six sentences. The final score is calculated as the SNR Loss score (in dB) using the correctly repeated keywords from the presented sentences with the following equation: SNR Loss score = 25.5 – (# of words correct). The SNR Loss score indicates the dB increases in SNR required by a hearing-impaired person to understand speech in noise, compared to normal hearing listeners. Therefore, the smaller the SNR Loss, the better the participant performed.

#### EEG Recording

Electroencephalographic recordings were administered using the Neuroscan system (NuAmps, Compumedics Neuroscan, Inc., Charlotte, NC, United States) with a 40-electrode cap in accordance with the International 10–20 system. The stimuli, created in a similar way as the stimuli in the FCDT test, were 1-s 0.25 kHz tones containing an upward frequency change of 0%, 10% or 50% at 0.5 s after the tone onset. Due to time constraints, only one base frequency (0.25 kHz) was used to minimize EEG testing time (approximately 1 h including the time for EEG cap placement and EEG recordings) for the participants. The 0.25 kHz is mapped to the first electrode in Cochlear devices and it is within the range of fundamental frequencies of human voice. CI users were presented with 400 trials for each type of the three stimuli (0%, 10%, and 50% change), with the inter-stimulus-interval at 0.8 s. The order of the stimuli was randomized. During testing, participants were instructed to avoid excessive eye and body movements. Participants read self-selected books or watched movies with closed captioning to keep alert and were asked to ignore the acoustic stimuli.

#### Music Training

The participants used a music streaming program (i.e., Pandora) downloaded onto personal electronic devices for training. The participants’ primary focus during training was the melody rather than other musical elements such as the rhythm (related to temporal features of the music) and timbre (allows the differentiation of sounds from different instruments). The focus was on melody only because: (1) melodies contain rich dynamic frequency changes; (2) CI patients have difficulties in pitch-based tasks such as melody; (3) cortical processing of music elements differ between melodies and other elements and training with the melody only would minimize the confounding effects from other music elements on cortical responses (Vuust et al., 2012; Lappe et al., 2013). The participants listened to music genres and materials of their choice that have an emphasis on melody. They were given the information about music melody and the opportunities to ask any questions they had before the training. Genres such as rap or hip-hop were excluded because the main musical component is speech or rhythms rather than melody. The training was performed at a comfortable loudness level via a personal audio cable or Bluetooth streaming. Training was completed during a distraction-free period of the day. The training regimen was 40 min/session/day × 5 days/week × 4 or 8 weeks. The selection of 4- or 8-week training was based on personal preference. The participants were required to log time spent training daily, what music genres were used, and which days in a week they performed the training.

### Data Analysis

For this study, the non-EEG data included the SSQ scores, hearing thresholds, FCDTs, CNC and AzBio scores in percent correct, and QuickSIN SNR Loss scores. The CAEPs were obtained from the EEG data using the following procedures of data processing.

Continuous EEG data were digitally filtered using a band-pass filter (0.1 to 30 Hz) and segmented into epochs from −100 to 1000 ms surrounding the stimulus onset. Following baseline correction, data were then imported into the EEGLAB toolbox (Delorme and Makeig, 2004) running under MATLAB (Mathworks Inc.) for further analysis. Visual inspection of the data epochs which contained non-stereotyped artifacts were removed.

Independent component analysis (ICA, Delorme and Makeig, 2004) was performed for the identification and removal of artifacts including eye-movement and CI artifacts. The method of using ICA for artifact removal is described in detail in EEGLAB manual and our previously published papers (Delorme and Makeig, 2004; Zhang et al., 2009). After artifact removal, the datasets were reconstructed. Channels near the CI coil, which were not injected with recording gel, were removed and interpolated. Then the data were re-referenced to the common average reference. Finally, the data were averaged separately for each of the three types of frequency changes (0%, 10%, and 50% change) in each participant.

Further waveform analysis was performed using the mean responses from six electrodes (F3, Fz, F4, C3, Cz, and C4) in the fronto-central region where the response amplitudes for automatic auditory discrimination were the largest compared to electrodes in other brain regions (Roman et al., 2005). The advantage of averaging responses from multiple electrodes was that the variability in CI data could be reduced, as the final EEG waveforms include more trials (Roman et al., 2005). The wave peaks of the onset CAEP (N1 and P2 peaks) and ACC response (N1′ and P2′ peaks) were identified within their own latency ranges (approximately in 100–300 ms after tone onset for the onset CAEP and 600–800 ms after the tone onset for the ACC, respectively, see Liang et al., 2016, 2018).

### Statistical Analysis

For each participant, the dependent variables were hearing threshold (dB HL) at each test frequency, the FCDT (%) at each base frequency, the percent correct for the CNC word and AzBio in quiet tests, the SNR Loss (dB) value for the QuickSIN test, and the score for the SSQ test, and the EEG measures (the size/amplitude of the onset CAEP peaks and the ACC peaks). Descriptive statistics were computed for each dependent variable. The effects of music training on each of EEG results were analyzed using repeated measures analysis of variance (ANOVA). The Proc mixed procedure in the SAS statistical program (Statistical Analyses System, SAS Institute, Inc., Cary, NC, United States) was used; mixed models allowed for control of other related factors such as test frequency. For all comparisons, *p* < 0.05 was considered statistically significant. Tukey’s method (Shaffer, 1995) was used to correct for multiple comparisons for factors with more than two levels. Because the training results showed large variability in non-EEG data, the results from non-parametric tests (Wilcoxon Signed-Rank) were reported.

## Results

Of the total 11 participants who completed the music training program, 9 completed the 8-week training and two subjects completed 4-week training. The non-EEG data from all 11 participants and the EEG data from nine participants were available for analysis. The following are the music training effects, regardless of the training duration, on all measurements: hearing thresholds, CNC, AzBio, QuickSIN, and the CAEPs (onset CAEP and ACCs).

### Hearing Thresholds

**FIGURE 1:**
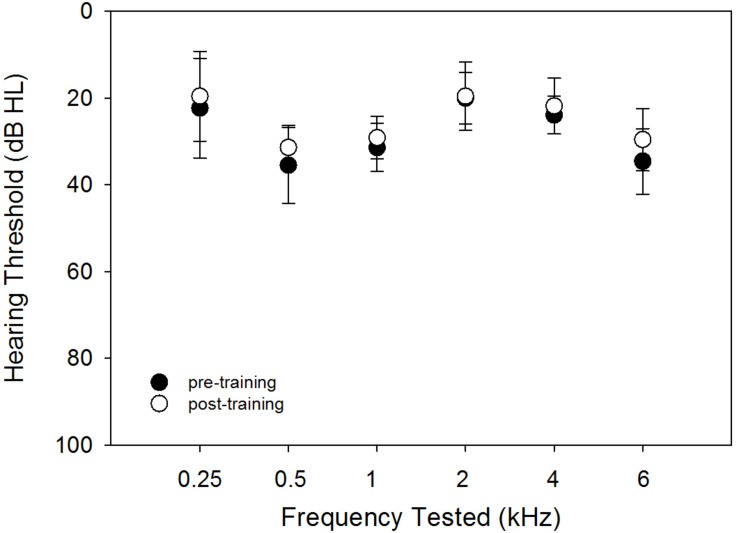
The pre- (solid circles) and post-training (open circles) hearing thresholds using pulsed tones at different frequencies. The means (circles) and the standard deviations (error bars) are plotted.

The hearing thresholds were tested using pulsed tones at frequencies of 0.25, 0.5, 1, 2, 4, and 6 kHz. Figure 1 shows the audiogram at pre- and post-training testing. This figure indicates that the precondition of audibility for the repeated measures of FCDT, CNC, AzBio, and QuickSIN was met. The small differences between the pre- and post-training thresholds may be related unknown variabilities such as microphone sensitivity, subject positioning, and the step size of 5 dB in the measurement protocol.

### Frequency Change Detection Threshold

**FIGURE 2:**
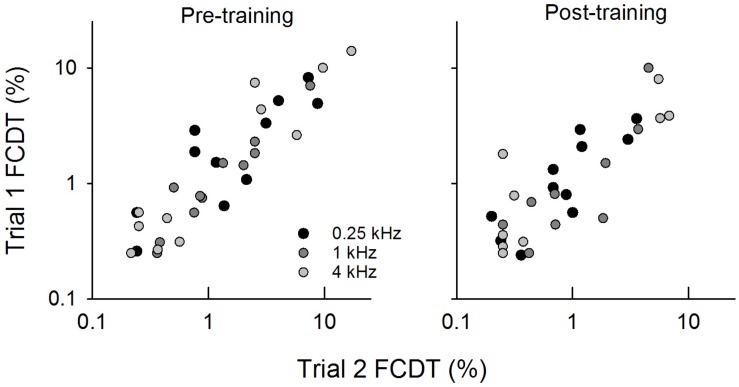
The scatterplots of FCDTs measured from trial 1 vs. trial 2 at pre- and post-training testing.

The frequency change detection tasks were tested twice during the pre- and post-training session to examine the test–retest reliability and reduce procedural learning effects. Figure 2 shows the scatterplots of the pre- and post-training FCDT results for trial 1 vs. trial 2. The difference between the pre- and post-training thresholds was analyzed using a repeated measures ANOVA, with Treatment (pre- vs. post-training) and the Base frequency as a fixed effect and the Subject and Trial number as a random effect. This model estimated the Trial number random effect to be zero, and therefore a simplified model with only the Subject as a random effect was conducted. Results showed that there was a significant effect for Treatment (*F* = 6.99, *p* = 0.0094). The FCDTs did not differ significantly between the three base frequencies (*F* = 2.34, *p* = 0.10).

**FIGURE 3:**
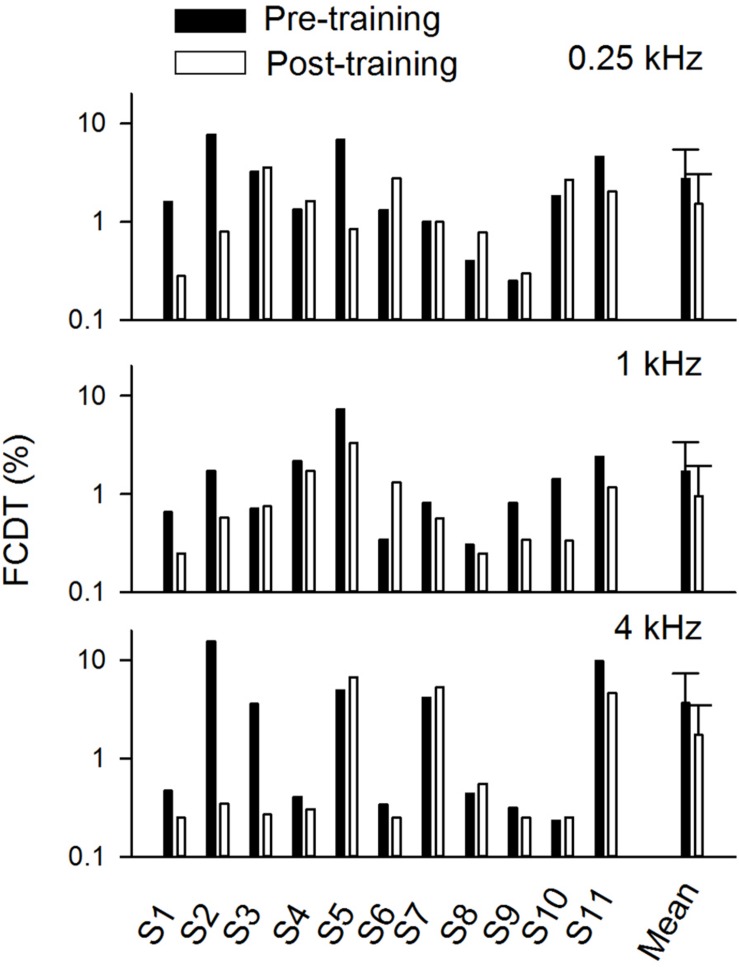
The pre- and post-training FCDTs at different base frequencies in individual subjects and the mean FCDTs of all individuals (*n* = 11). The error bar indicates 1 standard deviation.

Figure 3 shows the FCDT at pre- and post-training testing for the three tested base frequencies in individual CI users and the mean of all participants’ data. The averaged FCDTs at the two repeated trials were used for each participant. The mean FCDTs for pre-training vs. post-training were 2.74% vs. 1.52% at 0.25 kHz, 1.69% vs. 0.96% at 1 kHz, and 3.66% vs. 1.75% at 4 kHz.

### Speech Tests

**FIGURE 4:**
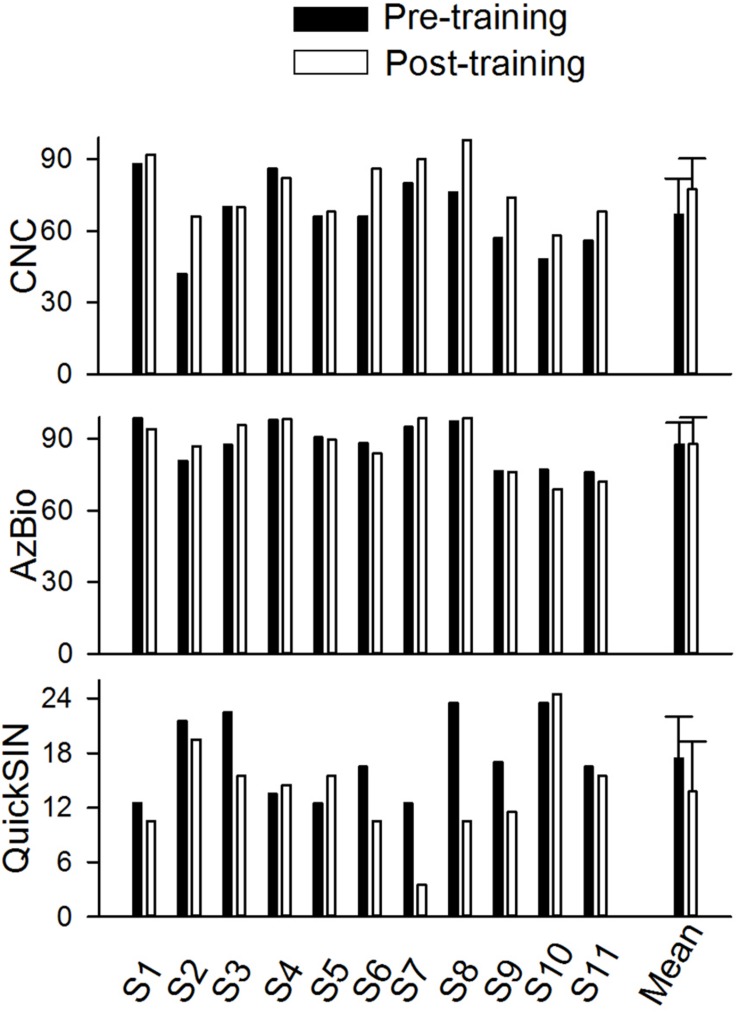
The pre- and post-training speech perception scores for CNC word (percent correct), AzBio sentence in quiet (percent correct), and QuickSIN (dB) in individual subjects and the mean values of all individuals (*n* = 11). The error bar indicates 1 standard deviation.

Figure 4 shows the speech performance at pre- and post-training testing in individual CI users and the mean of the data from the 11 trained participants. Mean CNC word recognition in quiet was 66.8% correct before training and 77.5% correct after training. Mean AzBio sentence recognition in quiet was 87.7% correct before training and 87.8% correct after training. The mean SNR Loss score for the QuickSIN was 17.5 dB before training and 13.77 dB after training. Pre- and post-training performance was compared for each speech test using a non-parametric Wilcoxon Signed-Rank tests. The results showed that speech performance significantly improved after training for the CNC (*p* = 0.0078) and QuickSIN tests (*p* = 0.039), but not for the AzBio test (*p* = 1.00).

### SSQ12

**FIGURE 5:**
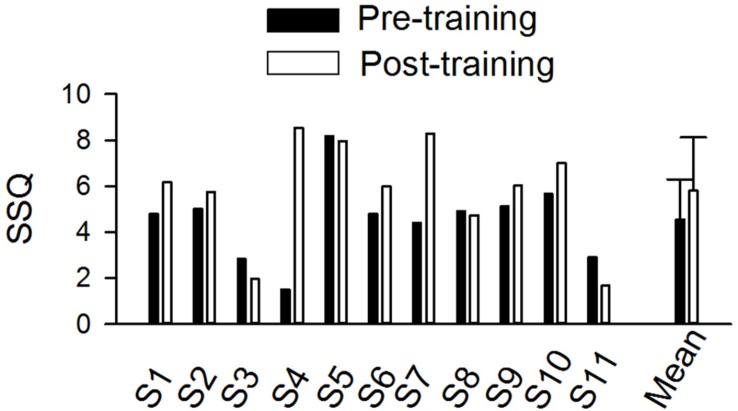
The pre- and post-training SSQ scores in individual subjects and the mean score of all individuals (*n* = 11). The error bar indicates 1 standard deviation.

Individual and mean scores on the SSQ12 are shown in Figure 5. The mean score for the SSQ12 improved after training (4.80 vs. 6.04). The difference between the pre- and post-training SSQ scores was analyzed the Wilcoxon Signed-Rank test, which showed no training effect on the SSQ (*p* = 0.10).

### Music Training on EEG Data

**FIGURE 6:**
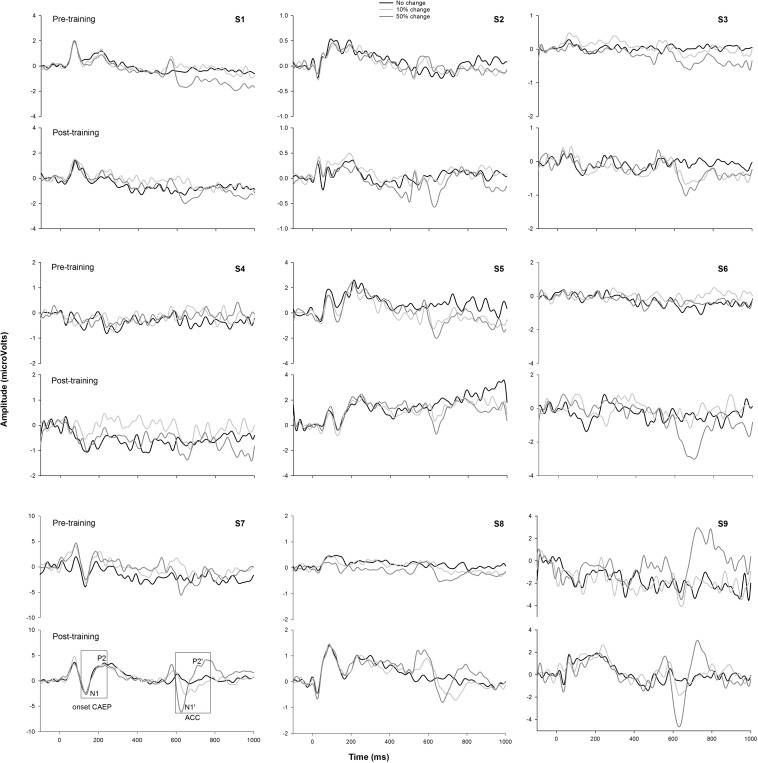
The pre- and post-training EEG data from the nine participants. Each trace represents the mean data from six electrodes (F3, Fz, F4, C3, Cz, and C4). The responses to the tones containing a 0% frequency change (black), 10% change (light gray), and 50% change (dark gray) are plotted. The onset CAEP peaks (N1 and P2) and ACC peaks (N1′ and P2′) are labeled in one subplot. Note that all participants finished a 8-week training except two participants (S2 and S9) who finished a 4-week training.

**FIGURE 7:**
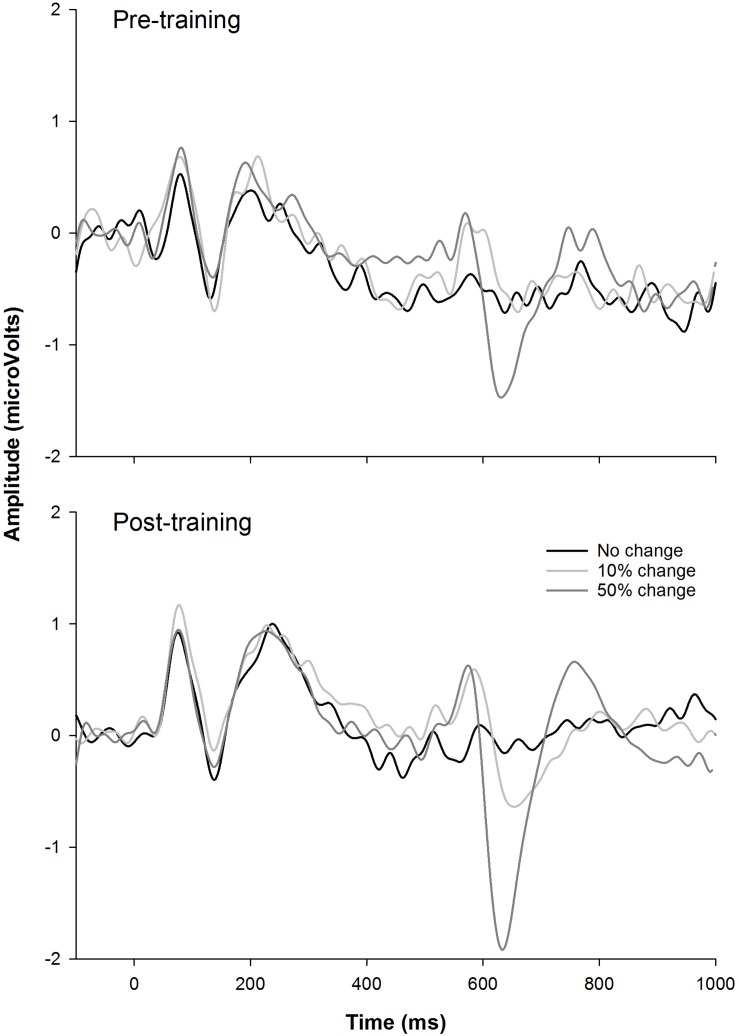
The mean EEG data from the nine participants. The responses to the tones containing a 0% frequency change (black), 10% change (light gray), and 50% change (dark gray) are plotted.

**FIGURE 8:**
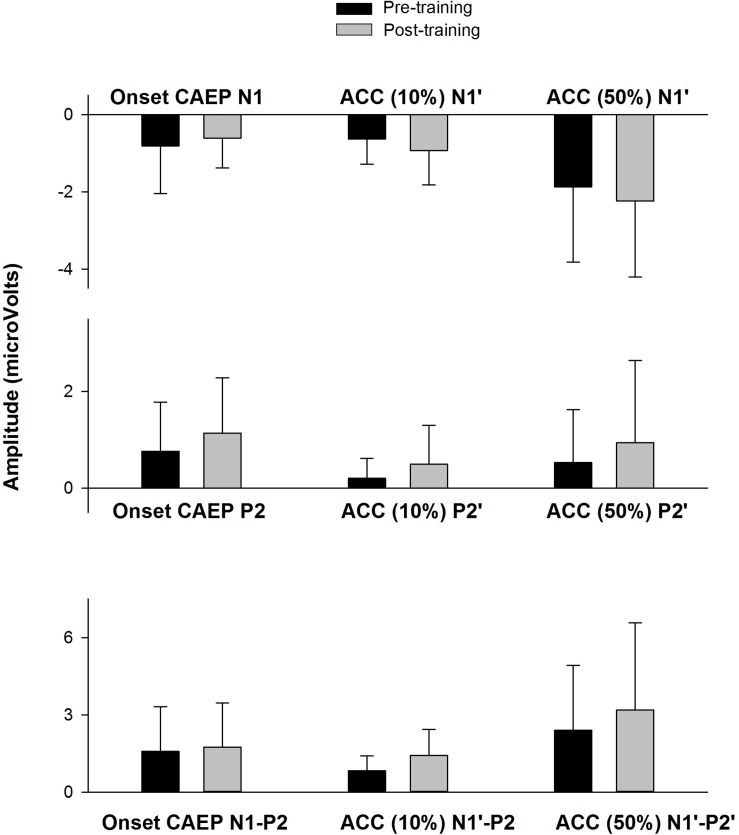
The means of the response amplitude at pre- and post-training testing (*n* = 9). The error bar indicates 1 standard deviation. The peak amplitudes (pre-training data in black, post-training data in gray) are displayed for the onset CAEP (N1, P2, N1–P2) and the ACC (N1′, P2′, and N1′–P2′) for the 10% and 50% change, respectively.

Figure 6 shows the CAEP waveforms at pre- and post-training testing in individual participants. In each plot, each waveform represented the data from six electrodes (F3, Fz, F4, C3, Cz, and C4) combined. The general trend observed is that post-training CAEPs are larger than pre-training CAEPs, with some participants showing more improvement than other participants. For instance, S1 and S5 showed no obvious improvement, while other participants showed improved response, with better morphologies and larger amplitude in the post-training CAEPs. Figure 7 shows the mean CAEP waveforms at pre- and post-training testing. Both the CAEP evoked by the tone onset and the ACCs evoked by the frequency changes (10% and 50% change) became larger after training. Figure 8 shows the means and standard deviations of the response amplitude at pre- and post-training testing.

Pre- and post-training EEG measures (peak amplitude) were analyzed using a mixed effects repeated measures ANOVA, with Treatment (pre- vs. post-training) and Response type (onset CAEP, ACC for 10% frequency change, and ACC for 50% frequency change) as fixed effects and Subject as a random effect. No significant effects for Treatment were observed for N1/N1′amplitude [*F*(1,38) = 0.10, *p* = 0.75], P2/P2′ amplitude [*F*(1,38) = 1.98, *p* = 0.17], or N1-P2/N1′–P2′ peak-to-peak amplitude [*F*(1,38) = 1.63, *p* = 0.21]. There were no interactions between Treatment and Response type for any of the EEG measures (*p* > 0.05).

## Discussion

In this study, questionnaire, behavioral, and EEG methods were used to examine the effect of short-term music training in CI users. Eleven of twelve participants enrolled completed the study, either with a 4-week training or 8-week training. A statistically significant improvement was found in FCDT, CNC words, and QuickSIN speech in noise perception. The onset CAEP and ACC showed improvement in morphologies and amplitudes in seven out of nine participants. However, the improvement in the response amplitude was not statistically significant (Figure 8), possibly due to the small sample size (*n* = 9) and the large variability in the CAEPs among participants.

The observed training effects cannot be explained with procedural learning due to the following reasons: (1) the procedural learning likely occurs at the beginning of the task when participants have difficulty remembering and executing the task but the performance quickly improves after they are familiarized with the task. This perceptual learning can be solved by providing a large number of trials to familiarize the participant with the task (Moore et al., 2003). In the current psychoacoustic task, there are 35 trials and subjects’ response are likely to reach the asymptotic performance after 1/3 of stimuli presented. Moreover, each participant was asked to complete the psychoacoustic task twice before the music training starts; the lack of significant difference between trial 1 and trail 2 indicates insignificant perceptual learning effects; (2) The CAEP, which is objective measure that shows strong test–retest reliability (Bidelman et al., 2018), showed clear improvements at post-training in most participants.

Note that procedural learning cannot be completely ruled out in the observed results, as some subjects exhibit better performance in trial 2 compared to trial 1 FCDT task for the baseline measures. To minimize the effects of procedural learning, all measures should have been measured until reaching asymptotic performance before training. In this study, the participants only performed the FCDT task twice and other tasks once at both pre- and post-testing sessions due to limited testing time. Therefore, potential procedural learning may have at least partially contributed to the observed training results.

The participants were trained using an attentive music listening program. The participants’ motivation was promoted through the following strategies: (1) asking the participant to complete training at home with music materials of their preference that have a focus on melody, (2) asking the participants to train their ears at the time of a day without distraction and log the training time and music genres, (3) letting the participants select the poorer ear for which they wanted to improve hearing, (4) letting the patient commit as long as they can in terms of training duration (4 weeks or 8 weeks), and (5) providing financial compensation for their time for training. As such, the training program had a relatively low attrition rate of 8.33%, with only 1 of the 12 enrolled participants dropping out of the study.

The attentive music listening program used for training in the current study is different from passive listening to the music one might do in daily life (e.g., doing irrelevant tasks during listening). Neurophysiological studies have reported different brain activations between attentive and passive listening conditions with music materials (Jancke et al., 2018). The differences between passive vs. attentive listening can also be seen in the cortical responses to an oddball paradigm that consists of rarely presented stimuli (deviants) and frequently presented stimuli (standard stimuli). Specifically, while the passive listening during an oddball paradigm elicits pre-attentive auditory evoked potentials (e.g., MMN, orienting response P3a), attentive listening during an oddball paradigm can elicit P3b, which involves auditory attention.

The attentive music listening training used in this study, compared to the training using relatively simple sound discrimination tasks, more likely targets on the top-down processes (e.g., attention and memory) that are essential for perceptual learning. While the bottom-up approach may improve performance within the trained domain effect, top-down music training may generalize to non-trained domains and positively affect speech outcomes (Fuller et al., 2018). Such enhanced top-down processing may be the reason for the improved speech performance and hearing sensitivity to frequency changes observed in this study. It would be interesting to examine if other sorts of training focusing on improving the top-down processing also result in the improvement in speech outcomes in future studies.

The current results showed that the CNC word recognition test and QuickSIN were improved significantly after training. There was a large variance in the change of CI outcomes after training; nine out of eleven participants showed improvement of 2–24 percentage points on the CNC word test and seven out of eleven participants had an improvement of 2–13 dB on the QuickSIN. The lack of statistical significance in the SSQ was likely due to the small sample size (*n* = 11) or the minimal effect on the patients’ self-evaluation of hearing, which may reflect more of real-world listening experience but is more affected by subjective expectation compared to the speech tests and EEG tests in the research lab. The lack of improvement in AzBio sentence in quiet is likely due to the ceiling effects of AzBio sentences in quiet, with high scores even before training (mean greater than 85%). Here one example of a subjects’ description of training effects is quoted: “I believe I can hear better with my left (weaker) ear than I could before the study. Some sounds that were previously in the background are more recognizable now. Previously I could not hear those sounds or they were unrecognizable as buzzing or static.”

The lack of statistical significance in the improvement in CAEP amplitudes may be related to the large variability among subjects and the small sample size (*n* = 9) involved in the analysis. However, it is worth noting that most participants (seven out of nine) displayed improved CAEP morphology and amplitude following training. Moreover, the participants with the most improvement in performance on speech tasks (e.g., QuickSIN, S3, S6, S7, S8, and S9 in Figure 4) showed more prominent improvement in the CAEP data (Figure 6) than those displaying less improvement or no improvement in speech performance. This finding may indicate that the improvement in speech outcomes following music training is related to the improved cortical encoding of sound features (including stimulus onset and the within-stimulus frequency changes).

Overall, short-term music training focusing on attentive music listening improves frequency change detection ability, word recognition, and speech perception in noise. It is believed that the speech perception in speech noise in the QuickSIN represents auditory functions at more central levels beyond cortical coding (Cullington and Zeng, 2008). Patel (2014) have explained the music effects on speech domain using an OPERA (Overlap brain networks, Precision of neural processing of sound features, and Emotion brought by music training, Repetition of the training, and Attention) hypothesis. Specifically, music places higher demands on sensory and cognitive components of perceptual learning than speech; with these higher demands, the emotion rewards of music, the frequent repetition of music training, and the focused attention, neural changes in the auditory system would occur to promote speech processing.

Note that CI users do not perceive the pitch changes the same way as normal-hearing listeners and the exact cue used by CI users for pitch perception is still under debate (Oxenham, 2008, 2013). Studies have indicated the frequency change cues and melody information may be changed into temporal (intensity or loudness changes) and place cues (electrode changes) by the speech processing strategy (Cousineau et al., 2010; Zhang et al., 2019). Such evidence is also shown in the electrodogram of the tones containing frequency changes in our recent publication (Zhang et al., 2019). Regardless of the cues used, music training appears to enable CI users to better detect these cues through improved neural encoding, as evidenced by the improved EEG results in most participants.

### Limitations and Future Studies

First, a limitation of this study is the small sample size, variability in training outcomes, and the use of monetary compensation that may have led to participants’ bias in the questionnaire results. Second, results may have been affected by confounding factors such as the difference in music materials, training duration, motivation level, etc. Third, there is a lack of control intervention, which is important to examine effects of procedural learning (Jiam et al., 2019). Fourth, attentive listening appears to be feasible for adult patients, but it is a challenge for pediatric patients, who may need familial involvement and more interesting training methods to boost patients’ engagement (Driscoll et al., 2015). Finally, the FCDT test only involved pure tones rather than complex stimuli. Future studies will use a larger sample size with more homogenous group, training methods that can increase patients’ engagement and motivation, more strictly guided music listening materials, control training program, and testing stimuli that reflect sounds in the environment such as music and speech.

## Conclusion

This study showed positive effects of a short-term music training program on speech perception, the ability to process frequency changes, and the cortical processing of sounds in some CI users. As the current research design may result in the potential contribution of procedural learning effects to the training effects observed, future studies will use a better design such as a randomized controlled crossover design to accurately examine the effects of perceptual learning following music training in CI users.

## Data Availability Statement

The datasets generated for this study are available on request to the corresponding author.

## Ethics Statement

The studies involving human participants were reviewed and approved by the Institutional Review Board of the University of Cincinnati. The patients/participants provided their written informed consent to participate in this study.

## Author Contributions

GF performed the experiments, data analysis, and drafted the manuscript at the University of Cincinnati. FZ designed and helped with experiments, analyzed the data, and revised and finalized the manuscript. CB, CL, KM assisted FZ and GF in subject recruitment and experiment implementation. JX contributed to experimental design and manuscript preparation. NZ helped with data statistical analysis and manuscript preparation, contributed equally to this work. All authors discussed the results and implications and commented on the manuscript at all stages.

## Conflict of Interest

The authors declare that the research was conducted in the absence of any commercial or financial relationships that could be construed as a potential conflict of interest.

## Footnotes

1 http://angelsound.tigerspeech.com/
